# A retrospective review of pain management in Tasmanian residential aged care facilities

**DOI:** 10.3399/bjgpopen18X101629

**Published:** 2019-03-06

**Authors:** Felicity Veal, Mackenzie Williams, Luke Bereznicki, Elizabeth Cummings, Tania Winzenberg

**Affiliations:** 1 Lecturer in Pharmacy Practice, Unit for Medication Outcomes Research & Education (UMORE), Pharmacy, School of Medicine, University of Tasmania, Hobart, Australia; 2 Lecturer in Pharmacy Practice, Unit for Medication Outcomes Research & Education (UMORE), Pharmacy, School of Medicine, University of Tasmania, Hobart, Australia; 3 Professor in Pharmacy Practice, Unit for Medication Outcomes Research & Education (UMORE), Pharmacy, School of Medicine, University of Tasmania, Hobart, Australia; 4 Senior Lecturer, Nursing and Midwifery, School of Health Sciences, Hobart, University of Tasmania, Hobart, Australia; 5 Professor in Chronic Disease Management, Menzies Institute for Medical Research, University of Tasmania, Hobart, Australia

**Keywords:** Pain, Prescribing, Care of the elderly, Patient groups, general practice, primary healthcare

## Abstract

**Background:**

The management of pain by GPs for residents of aged care facilities (ACFs) is very common.

**Aim:**

To measure the prevalence and assess the management of pain in ACF residents, particularly those with dementia.

**Design & setting:**

A retrospective review of ACF residents’ medical records was undertaken at five southern Tasmanian (Australia) ACFs.

**Method:**

Data extracted included results of the most recent assessment of pain and its management, frequency and treatment of pain incidents in the previous 7 days, demographics, and medical and medication history. Univariate analysis was used to identify variables associated with increased frequency of pain episodes.

**Results:**

The final analysis included 477 residents. At least one episode of pain in the preceding 7 days was documented in 25.6% (*n* = 122) of residents' notes. Pain episodes were most commonly managed by analgesics (45.5%), massage (40.7%), and heatpacks (13.8%). Residents with dementia were not less likely to have pain identified during the past week (14% versus 20%; *P* = 0.09), but they were much less likely to have pain identified on their most recent pain assessment (*P* = 0.03).

**Conclusion:**

GPs should carefully consider the suitability of using ‘as required’ analgesics. Furthermore, on admission to an ACF, GPs need to ensure a patient’s medical history includes all pain or potentially pain-causing conditions, to ensure that a resident’s pain assessment is complete. This is especially necessary for those with dementia, to ensure that staff remain vigilant about the possibility of the resident experiencing pain.

## How this fits in

Pain in nursing home residents is prevalent and potentially suboptimally managed, with 17.5% of residents reporting pain ≥2 times a week being prescribed only ‘as required’ analgesics. To ensure optimal pain management is maintained, clinicians need to ensure that residents’ pain management is dynamic and adjusted to the current pain experience.

The utilisation of non-pharmacological therapies was common, but further research is required to determine how these should be used to provide the most benefit.

The identification of pain on the pain assessment form for residents with dementia was lower. It is imperative that resident’s medication history prior to admission to an ACF includes all pain or potentially pain-causing conditions.

## Introduction

The prevalence of pain increases with age,^1,2^ with >80% of ACF residents experiencing persistent pain.^3^ The assessment and management of pain for residents of ACFs is complicated by many factors including: a lack of clinical trial evidence to support pharmacological and non-pharmacological therapies (NPTs);^4–6^ changes to pain perception;^7–9^ a reluctance to report pain or take analgesics;^10,11^ and an increased likelihood of experiencing adverse events or interactions with analgesics.^4,12,13^ Additionally, for residents who cannot communicate effectively, pain assessment relies on using observation tools or informal observation, which have been found to have variable accuracy in identifying pain,^14,15^ often resulting in suboptimal pain management, particularly in those with dementia.^16–18^

There are also a number of institutional challenges in Australia, as well as internationally, which complicate pain management including time pressures (for both nursing staff and GPs); inadequate consultation space for GPs to review patients; the use of agency nurses reducing continuity of care; low levels of staff training and pay; communication barriers between nursing staff and GPs; and insufficient remuneration and time allocated for GPs to manage ACF residents.^19–22^ There is also a common practice in Australia of pharmacies providing GPs with lists of prescriptions that are needed for continued dispensing of residents’ dosage administration aids (known as monitored dosage systems in the UK),^23^ rather than normal practice of reviewing the patient when new prescriptions are required. Due in part to all of these challenges, studies have frequently indicated that pain is often suboptimally treated in ACFs.^24–26^

The authors previously reported on pain management in ACFs using information recorded during formal medication reviews undertaken by pharmacists.^27^ Reviewing 7309 medication profiles of ACF residents throughout Australia between 2010–2012 demonstrated that analgesic use was high (91%), with nearly one-third of residents prescribed regular opioids (29%), but the study was limited in its ability to ascertain the suitability of the prescribed analgesics, as pain frequency was not available for the residents. Consequently, the primary aims of the present study were to measure the prevalence of pain in Tasmanian ACF residents, assess how pain was managed (including analgesics and NPTs), and examine resident characteristics associated with increased frequency of pain episodes. Additionally, due to the previously identified suboptimal management of pain in patients with dementia, this review will also specifically examine the impact of dementia on documentation and management of pain.

## Method

All ACFs in southern Tasmania, Australia with dementia and non-dementia specific beds were contacted to participate in this study (*n *= 16). Data collection was undertaken between September– October 2015; this was undertaken by FV, MW, and SR (research assistant). Only permanent residents of the ACFs were included in this study, with all short-term respite residents excluded. Medical records were reviewed, and the data extracted included resident demographics, medical and medication history, information from their most recent pain assessment (see below), and whether the resident had reported or been identified as experiencing pain in the past 7 days and, if so, how this was treated. This was based on notes written by the nursing staff and/or documentation of the use of ‘as required’ analgesics. ACF staff were interviewed at the time of conducting this review regarding pain management and assessment, and it was noted that pain was frequently informally reviewed and residents would be assessed whenever pain was noted or suspected in residents.^21^

As part of the Australian Aged Care Funding Initiative (ACFI), residents must undergo a regular assessment of their pain and its management.^28^ These assessments are carried out by the ACF staff, generally a registered nurse, on admission to the ACF and thereafter ideally every 3 months and/or when there is a change in the health status.^29^ Information from the resident’s most recent pain assessment was also collected including the site(s) of pain, the cause(s), and prescribed treatment modalities.

Each resident who was recorded as taking an opioid regularly had their average daily oral morphine equivalence calculated. For ‘as required’ opioids, the calculation was based on the number of ‘as required’ opioid doses they had received in the preceding 7 days. The Charlson Comorbidity Index (CCI),^30^ which estimates 1-year mortality based on comorbidities, was calculated for each resident to assess level of morbidity burden. This measure was chosen so that comparisons could be made with the previous study.^27^

Analysis was performed using SPSS Statistics for Windows (version 20). Univariate analysis was undertaken using χ^2^ and Mann–Whitney U tests to evaluate differences between those who had a history of dementia and those who did not. Consent was obtained from each facility manager to review and record de-identified resident information.

## Results

Five of the 16 ACFs approached agreed to participate within the timeframe available for study implementation. The ACFs reviewed were high care facilities (nursing homes), although one facility also had some low-dependency beds (residential homes). The median number of beds per facility included in the analysis was 99 (range 85–171).

Records of 496 residents were reviewed. Eighteen were excluded because they either managed their medications themselves or because their medication charts were not available, and one resident was excluded because they died in the preceding week. The final analysis included 477 residents. The median age was 85.1 years (interquartile range [IQR] 80.1–91.2), the median CCI score was 2 (IQR 1–3), and 65.3% of the residents were female. The majority of residents (*n* = 382; 80.1%) were documented as experiencing pain on their most recent ACFI pain assessment. Table 1 shows the analgesics prescribed on the medication chart to the residents of the ACFs. Analgesics were prescribed to 91.8% of residents, with paracetamol being the most commonly prescribed analgesic. Table 2 shows the most common causes of pain and the recommended treatment modalities from the ACFI pain assessment. Musculoskeletal pain was most frequently noted as a cause of pain in residents, with analgesics being recommended for 70.6% of the residents, followed by NPTs, particularly heatpacks (49.7%) and massage (43.2%).

**Table 1.** Pain management strategies prescribed to the ACF population (*n* = 477)

Analgesic	*n* (%)
Any analgesic	438 (91.8)
Regularly dosed analgesic	305 (63.9)
As required analgesics	321 (67.3)
Both as required and regularly dosed analgesics	188 (39.4)
Only as required analgesics	131 (27.5)
Regularly prescribed paracetamol	272 (57.0)
As required paracetamol	212 (44.4)
Oral non-steroidal anti-inflammatories, regularly dosed	16 (3.4)
Oral non-steroidal anti-inflammatories, as required	13 (2.7)
Tricyclic anti-depressant	14 (2.9)
Gabapentinoids	40 (8.4)
**Regularly prescribed opioids^a^**	139 (29.1)
Buprenorphine patch	73 (52.5)
Extended release oxycodone/naloxone	28 (20.1)
Weak opioids (tramadol; paracetamol with codeine)	17 (12.2)
Immediate release oxycodone	11 (7.9)
Extended release oxycodone	8 (5.8)
Extended release morphine	5 (3.6)
Fentanyl patch	5 (3.6)
Morphine in a syringe driver	4 (2.9)
Immediate release morphine liquid	2 (1.4)
Hydromorphone	2 (1.4)
Tapentadol	1 (0.7)
**As required opioids^a^**	169 (35.4)
Immediate release oxycodone	68 (40.2)
Morphine sulphate injection	38 (22.5)
Paracetamol with codeine	36 (21.3)
Morphine liquid	19 (11.2)
Codeine phosphate	12 (7.1)
Tramadol	11 (6.5)
Hydrocodone	4 (2.4)
Extended release oxycodone/naloxone	1 (0.6)
Fentanyl injection	1 (0.6)

^a^Percentage does not equal 100% because some residents were prescribed more than one regular opioid or 'as required' opioid.

**Table 2.** Cause of pain and treatment modalities prescribed on ACFI assessment

	*n* (%)
**Cause of pain (*n* = 382)**	
Musculoskeletal, lower extremities Back or neck pain Musculoskeletal, upper extremities Generalised joint pain Visceral pain Neuropathic pain Post-fracture/osteoporosis pain Inflammatory joint conditions Headache/migraine Cancer-related pain	191 (50.0) 134 (35.1) 112 (29.3) 32 (8.4) 17 (4.5) 15 (3.9) 13 (3.4) 13 (3.4) 11 (2.9) 3 (0.8)
**Treatment plan (*n* = 477)^aa^**	
Analgesics Heatpacks Massage Repositioning Rest Gentle exercise/physiotherapy programme One-on-one calming Elasticated garment Technical equipment, for example TENS	337 (70.6) 237 (49.7) 206 (43.2) 151 (31.7) 117 (24.5) 90 (18.9) 47 (9.9) 12 (2.5) 11 (2.3)

^a^Residents were often documented as having a pain treatment plan even if there were no documented pain causing condition

Most (74.4%) residents did not report, or were not observed as, experiencing pain during the preceding 7 days: 8.4% of residents experienced 1 episode; 8.0% experienced 2 episodes; and 9.2% experienced ≥3 episodes of pain. The most common forms of management of painful episodes were the provision of analgesics (45.5%), massage (40.7%), and heatpacks (13.8%). ‘As required’ only analgesics were prescribed to 17.5% of residents who experienced pain ≥2 times in the past week. There was also no difference between the use of ‘as required’ only analgesics in those who experienced ≥2 painful episodes with or without dementia (16.7% versus 17.3%; *P* = 0.94). Table 3 shows the characteristics of the residents with and without dementia. This shows that the documentation of pain-causing conditions, as well as documentation of treatment options on the ACFI form, are less likely to occur for residents with dementia.

**Table 3.** Baseline demographics and patient characteristics of those with and without dementia

	Residents with dementia, *n* (%)(*n* = 215)	Residents without dementia, *n* (%)(*n* = 262)	*P* value
Mean age, years (IQR)	85.7 (79.7–90.6)	87.3 (80.9–91.5)	0.07
Female	80 (48.5)	177 (56.9)	0.26
Mean number of regular medicines (IQR)	7 (5–10)	9 (6–12)	<0.01
**ACFI pain assessment**			
ACFI pain-causing condition	163 (75.8)	219 (83.6)	0.03
ACFI analgesics	135 (64.3)	193 (74.2)	0.02
ACFI NPT	152 (70.7)	211 (80.5)	0.01
**Medication chart**			
Any analgesia	199 (92.6)	239 (91.2)	0.60
Regularly prescribed	137 (63.7)	169 (64.5)	0.86
As required only	61 (28.4)	70 (27.7)	0.69
RD paracetamol	121 (56.3)	151 (57.6)	0.77
RD opioid	57 (26.5)	83 (31.7)	0.22
MEQ RD opioid, mg (IQR)	0 (0–6)	0 (0–10)	0.22
**Pain experience**			
≥2 pain experiences per week	30 (14.0)	52 (19.8)	0.09

IQR = interquartile range. NPT= non-pharmacological therapy. MEQ = morphine equivalence. RD = regularly dosed.

## Discussion

### Summary

This study adds significantly to the understanding of how pain is managed in ACFs and indicates that the majority of residents have good pain management. Overall, this study has three key findings: firstly, that the use of NPTs is common when pain is identified; secondly, that a significant proportion of residents (both with and without dementia) are receiving potentially suboptimal pain management, especially in relation to the use of ‘as required’ analgesics; and thirdly, that the documentation on the ACFI pain assessment identified fewer pain-causing conditions and were less likely to recommend use of NPTs and analgesics to residents with dementia, potentially indicating a reduced identification of pain-causing conditions in this population.

### Strengths and limitations

This study has a number of strengths, such as the comprehensive data collection, including all medications taken in the preceding 7 days as well as the number of documented pain instances. Although it is unlikely, the authors are unable to be certain that there are not systematic differences between the ACFs that chose to participate and those that did not. A limitation of this study was the small geographic location in which the study was conducted, and small number of included ACFs. The results are comparable to a larger Australian study,^27^ however, in relation to demographics and analgesic use; thus, the results are likely to be generalisable to the wider Australian ACF population. Due to differences between Australian and international management, however, the generalisability to non-Australian ACFs is unknown. The methodology of a cross-sectional retrospective review has a number of limitations and the potential for bias but, due to the high number of residents who experience pain in this population, it is likely that the results are generalisable to the wider ACF population.

It is possible that this study underestimates the prevalence of pain in ACF residents, as it is likely that a number of residents experienced discomfort and pain and were repositioned, and these incidents were not documented in the notes. More clinically important persistent or intense pain requiring more active treatment are captured, however. The other factor that was unable to be accounted for in this study was the effect of regularly provided NPTs, such as massage; it is unclear whether these were consistently provided to the residents or not, and the impact that these have.

### Comparison with existing literature

Undermanagement of pain in residents with cognitive impairment has been frequently noted in the literature.^17,18,27^ Positively, this study did not find a significant difference between the management of those with and without dementia in relation to overall analgesic use (Table 3), as is supported by another recent Australian study.^15^ However, there was a lower rate of documented pain conditions and prescribing of pharmacological therapies and NPTs as part of a pain management plan on the ACFI pain assessments of patients with dementia. This may indicate that, although treatment did not vary, there is still underrecognition and poor documentation of pain in residents with dementia. It is very important that a resident’s medical history is as complete and up-to-date as possible when entering ACFs, to ensure that any potential or actual pain-causing condition that these residents may have is noted, and to reduce the likelihood of missed or underidentified pain.

In addition, for residents with dementia, guidelines recommend by-the-clock dosing^6^ to help overcome the poor rates of identification of pain in this population. In this study, the use of regular and ‘as required’-only analgesics was similar in residents with and without dementia. Because residents with dementia cannot always express their pain, staff must be alert to the signs of pain. Potentially this alertness could be reduced if residents are 'known' not to have pain, based on their ACFI pain assessment. Further research is required to understand why the documentation of pain is substantially lower than in patients who have dementia, and to develop and test strategies to reduce this underreporting.

In those residents who experienced pain ≥2 times in 1 week, there was a high reliance (27.5%) on ‘as required’-only analgesics, in residents both with and without dementia. Moreover, 17.5% of residents who experienced ≥2 pain incidences in the past week were only prescribed ‘as required’ analgesics. It is possible that the use of ‘as required’ medication may indicate judicial use of analgesics; for example, if the pain is sporadic in its presentation and/or mild in intensity, or if prescribers are trying to limit the exposure to opioids due to concerns regarding side effects in this population. However, it may be that this is indicative of suboptimal pain management in situations where frequent episodes are a marker of poor pain control and/or regular analgesia has not been considered. This is an area that requires further research to identify the rationale behind the prescribing of ‘as required’ analgesics in this population, to ensure that optimal prescribing and management occurs.

Suboptimal pharmacological pain management may be worsened by the common practice of GPs being provided lists of prescriptions required for dosage administration aids, rather than the standard practice in the community of being reviewed by a GP when a new prescription is required. This model of care has the potential to result in a situation where analgesic regimens may not be sufficiently assessed on a regular basis, leaving residents on inadequate or inappropriate analgesic regimens. This is a possible contributing factor to the high use of ‘as required’ medications in those experiencing ≥2 pain episodes a week.

As noted previously, the frequency of use of non-pharmacological management strategies has been poorly documented^31^ in the literature with most studies reviewing analgesic use alone. This is despite a number of guidelines recommending the use of NPTs in older people.^32,33^ This study found that the use of NPTs in addition to pharmacological interventions was commonly included in pain management plans, as recommended by best practice guidelines,^6,32^ though the frequency of the actual delivery of the various NPTs individually or in combination is not known. However, it is still unclear how NPTs are best utilised, with trials often being of poor quality, although demonstrating some benefit.^34–36^ This is an area that requires further research to ensure that residents are receiving optimal care and the use of NPT is suitable. Further research as to how best engage residents in gentle movement exercises is also needed to support the use of NPTs alongside analgesics.

### Implications for research and practice

This study has important implications for both research and practice. It has been noted in previous studies^21,37^ that the documentation of pain in ACF nursing notes is not complete, but medication provision must be documented. Thus, potentially, the use of ‘as required’ analgesia beyond a certain level could be treated as a trigger for reassessment of pain management strategies by ACF staff, GPs, and pharmacists in a multidisciplinary approach. In cognitively impaired residents, GPs might need to take particular care to ensure accurate recording of existing pain conditions, and be vigilant with regards to accurate identification of painful episodes and the need for regular (as opposed to ‘as required’) analgesia. Further research could investigate the effect of more frequent one-to-one assessment by the GP, nurse practitioner, pharmacist, or ACF nurses on pain frequency, appropriateness of pain management, functionality, and quality of life.

In addition, the evidence base for NPTs needs to be strengthened. In particular, the effectiveness of NPTs in ACF residents needs further research to ensure that the way in which residents are managed with NPTs is beneficial and undertaken optimally. In conclusion, a multifaceted approach to improving pain management in ACFs is needed. To ensure optimal pain management is maintained, the following steps should be taken: (a) clinicians need to ensure that residents' pain management is dynamic and adjusted to the current pain experience; (b) skills need to be improved in ACF staff for identifying pain in potential at-risk groups especially when ‘as required’-only analgesics are prescribed; and (c) research is needed to generate further evidence to support the use of both NPTs.
